# Low Frequency Groans Indicate Larger and More Dominant Fallow Deer (*Dama dama*) Males

**DOI:** 10.1371/journal.pone.0003113

**Published:** 2008-09-03

**Authors:** Elisabetta Vannoni, Alan G. McElligott

**Affiliations:** Zoologisches Institut, Universität Zürich, Zürich, Switzerland; University of Sussex, United Kingdom

## Abstract

**Background:**

Models of honest advertisement predict that sexually selected calls should signal male quality. In most vertebrates, high quality males have larger body sizes that determine higher social status and in turn higher reproductive success. Previous research has emphasised the importance of vocal tract resonances or formant frequencies of calls as cues to body size in mammals. However, the role of the acoustic features of vocalisations as cues to other quality-related phenotypic characteristics of callers has rarely been investigated.

**Methodology/Principal Findings:**

We examined whether the acoustic structure of fallow deer groans provides reliable information on the quality of the caller, by exploring the relationships between male quality (body size, dominance rank, and mating success) and the frequency components of calls (fundamental frequency, formant frequencies, and formant dispersion). We found that body size was not related to the fundamental frequency of groans, whereas larger males produced groans with lower formant frequencies and lower formant dispersion. Groans of high-ranking males were characterised by lower minimum fundamental frequencies and to a lesser extent, by lower formant dispersions. Dominance rank was the factor most strongly related to mating success, with higher-ranking males having higher mating success. The minimum fundamental frequency and the minimum formant dispersion were indirectly related to male mating success (through dominance rank).

**Conclusion/Significance:**

Our study is the first to show that sexually selected vocalisations can signal social dominance in mammals other than primates, and reveals that independent acoustic components encode accurate information on different phenotypic aspects of male quality.

## Introduction

Male vocalisations are often subject to sexual selection and can be used to assess the quality and condition of the caller in various vertebrates [1]–[4]. The reliability of the information encoded in sexually selected acoustic signals is maintained by constraints imposed on the caller, which make cheating costly [5]. Alternatively, vocalisations that are physiologically or physically constrained carry some characteristics that are directly related to intrinsic properties of the caller and therefore cannot be faked [6].

The relationships between the body size of callers and the acoustic parameters of vocalisations are of particular interest in species in which body size determines fighting ability and reproductive success [7]–[10]. In toads, frogs, and birds, body size is negatively related to the fundamental frequency of calls [11]–[13]. In mammals, and within a given species, body size is related to fundamental frequency across age categories and among adult females but not among adult males [9], [14]–[16]. Fundamental frequency corresponds to the rate at which the vocal folds of the larynx open and close and is determined by different factors such as the length of the vocal folds, longitudinal stress on the vocal folds, and the tissue density of the vocal folds [17]. For example, longer vocal folds result in lower fundamental frequency. Because the larynx is not constrained by the bones of the skull, the vocal folds may grow independently of the rest of the head or body [18]. Moreover, vocal folds are highly sensitive to changes in testosterone [19], [20] and they may grow longer in males with higher testosterone levels. Thus, fundamental frequency is a poor indicator of male body size in mammals.

Formant frequencies or resonances of the vocal tract probably represent the key acoustic variables linked to variation in body size in mammals [16], [18], [21]–[24]. Formants frequencies and their average spacing (formant dispersion) depend upon tissue structure, and the shape and length of the vocal tract [25]. Longer vocal tracts produce lower formant frequencies. In contrast to the vocal folds, the length of the vocal tract is constrained by skeletal structures (e.g. dimensions of the skull) and therefore closely tied to overall body size [18].

In addition to male body size, sexually selected calls might convey information about other indirect measures of male fitness, such as social dominance. Dominance rank and acoustic parameters can be indirectly related as they are both not fixed traits of the caller, but instead vary according to individual physical and physiological attributes [19], [26], [27]. The perception of these characteristics based on acoustic cues by competing males may affect the outcome of agonistic interactions. Females evaluating the relative quality of males might rely on acoustic cues related to dominance because high-ranking males often have better survival, and overall reproductive success than low-ranking males [7], [28], [29]. While there is now strong evidence that some acoustic parameters of vocalisations represent body size, the relationships between vocal parameters and other characteristics linked to male quality, such as dominance rank and mating success, have rarely been examined.

Fallow deer are ideal for investigating the role of acoustic signals as indicators of male quality. Fallow deer are characterised by a polygynous mating system with high male-male competition and skewed reproductive success [30], [31]. Larger males are generally higher ranked than smaller males, and rank is also closely associated with mating success [7]. Males only vocalise during the breeding season and the sexually selected call they produce is known as a groan [32], [33]. In the northern hemisphere, males start groaning approximately three weeks before the first matings take place (late September) and continue until the vast majority of matings have occurred (early November, [34]). Vocalisations are directed both towards males during agonistic encounters and towards females during chasing or herding behaviour, suggesting a potential role of groans in both male-male competition and female choice [35]. Vocalization rates appear to convey information to other males, and therefore to play a role in male mutual assessment [35]. However, acoustic components are salient to mammals and have a strong biological significance independent of the vocalization rate [36]–[38]. When males vocalise, the larynx is pulled down towards the sternum and the length of the vocal tract increases [39], [40]. As a consequence, the formant frequencies decrease and reach a minimum value that could reveal information related to male quality [16], [40]. The groans of fallow bucks are individually distinctive [41] and as is the case with red deer (*Cervus elaphus*), conspecifics are likely to discriminate between males based on the sound of their calls [42].

We investigated the relationships between the acoustic structure of fallow deer groans and male quality. We first determined whether body size is related to the fundamental frequency parameters, formant frequencies, and formant dispersion of groans. We then examined the relationships between the acoustic parameters (minimum fundamental frequency and minimum formant dispersion) and dominance rank and mating success, while also considering the role of body size.

## Results

### Relationships between Body Size and the Acoustic Parameters

**Figure 1:**
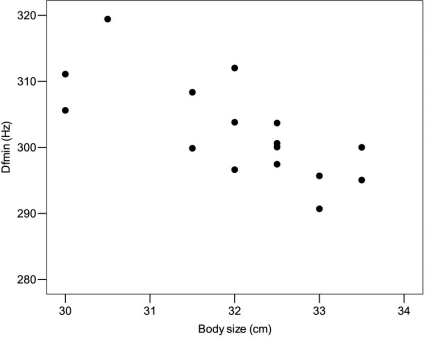
The negative relationships between body size and minimum formant dispersion (Dfmin). Bigger males emit groans characterised by lower minimum formant dispersion.

The segment of the hind leg that we used as indicator of male body size ranged from 30.0 to 33.5 cm (mean = 32.1±0.3, *N* = 17). Body size was not significantly related to fundamental frequency parameters (GLMM: F0min, F_1,15_ = 0.0002, P = 0.990, F0mean, F_1,15_ = 0.0462, P = 0.833, F0max, F_1,15_ = 0.0520, P = 0.823). With increasing body size, the first four minimum formant frequencies tended to decrease, but none of the relationships were significant (GLMM: F1min, F_1,15_ = 1.16, P = 0.298; F2min, F_1,15_ = 3.21, P = 0.094; F3min, F_1,15_ = 2.08, P = 0.171; F4min, F_1,15_ = 3.13, P = 0.097). There was a tendency for body size to be negatively related to the minimum frequency of the fifth formant (GLMM: F5min, F_1,15_ = 3.92, P = 0.066). Body size varied negatively with the minimum frequency of the sixth formant and the formant dispersion (GLMM: F6min, F_1,15_ = 4.58, P = 0.049; Dfmin, F_1,15_ = 18.93, P<0.001; Fig. 1).

### Relationships between Acoustic Parameters and Dominance Rank

**Figure 2:**
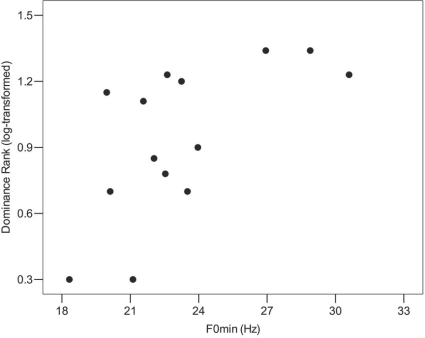
The negative relationship between F0min and dominance rank (log-transformed). Lower values of dominance rank indicate higher ranking males. Higher ranking males produced groans with lower minimum fundamental frequency.

Model selection favoured the model incorporating the minimum fundamental frequency (F0min), (lowest AICc; Table 1, model 2). This model shows that groans characterised by lower F0min are produced by higher-ranking males (Fig. 2: F_1,12_ = 8.43, P = 0.013). A close competitor of model 2 was model 4, which only included the minimum formant dispersion (Dfmin), (Table 1, Model 2 and 4: ΔAICc<2). The two models with the lowest AICc (model 2, 4) were together 64.8% supported by the data (combined Akaike weights, 0.443 and 0.205). However, the evidence ratio reveals that the model with the F0min (model 2) was more than twice as good as the second best model (model 4). The addition of Dfmin or body size to the best model did not result in a better supported model (Table 1, comparing model 2 with model 5: LRT χ^2^_1_ = 1.82, P = 0.18; comparing model 3 with model 2: LRT χ^2^_1_ = 2.27, P = 0.13). The model which includes only body size as a parameter was considerably less supported by the data (Table 1, model 13: <ΔAICc>7). Thus, F0min was the factor more strongly correlated with dominance rank. Dfmin is also related to dominance rank, but to a lesser extent. Body size was not related to rank.

**Table 1:** Results of the AIC model selection procedure used to investigate the relationships between acoustic parameters and dominance rank and mating success in male fallow deer.

Model		Log likelihood	K	AICc	ΔAICc	*w_i_*	Evidence ratio
First set of models:							
Dominance rank							
1	Body	−3.86	3	16.12	5.82	0.024	18.33
2	F0min	**−0.95**	**3**	**10.30**	**0.00**	**0.443**	**1.00**
3	Body+F0min	0.19	4	12.07	1.77	0.183	2.43
4	Df	−1.72	3	11.85	1.55	0.205	2.17
5	F0min+Df	−0.04	4	12.52	2.22	0.146	3.04
Second set of models:							
Mating success							
6	Body	−11.41	3	31.22	8.16	0.012	59.22
7	Rank	**−7.33**	**3**	**23.06**	**0.00**	**0.682**	**1.00**
8	Body+Rank	−6.73	4	25.91	2.85	0.163	4.17
9	F0min	−11.15	3	30.70	7.64	0.015	45.64
10	Df	−10.95	3	30.31	7.25	0.018	37.53
11	F0min+Df	−10.48	4	33.40	10.34	0.004	176.27
12	Body+F0min	−9.55	4	31.54	8.48	0.010	69.69
13	Rank+Dfmin	−7.27	4	26.98	3.93	0.096	7.11

Body size was also included as predictor in some of the models to check for its effect on dominance rank and mating success.

The fit of the models is assessed by Akaike's information criterion (AICc): the lowest value indicates the best fit (in bold). K is the number of estimated parameters included in the model. ΔAICc gives the difference in AICc between each model and the best model. The Akaike's weights (*w_i_*) assess the relative support that a given model has from the data, compared to other candidate models in the set. The evidence ratio is the ratio between the Akaike's weight of the best model and that of a competing one. This value is used to determine to what extent the best model is better than another. The covariates were: body size (Body), dominance rank (Rank), minimum fundamental frequency (F0min), and minimum formant dispersion (DFmin).

### Relationships between Acoustic Parameters and Mating Success

The model with only the rank included, was 61.9% supported by the data and clearly selected as the best model (lowest AICc; Table 1, model 7). This model was more than four times as good as the second best model in which body size was also included (model 8). The addition of body size did not significantly improve the best model (Table 1, comparing model 7 with model 8: LRT χ^2^_1_ = 1.19, p = 0.28). The models in which Dfmin, F0min were included together with rank, had considerably less support than the best model (Table 1, model 13: 3<ΔAICc<7). All other models were poorly supported by the data (Table 1, model 6, 9, 10, 11, 12). Thus, dominance rank appears to be the crucial factor which determines male mating success in fallow deer, with higher-ranking males having higher mating success (F_1,12_ = 8.43, P = 0.003). Any relationship between the acoustics parameters and mating success appears to be mediated by rank.

## Discussion

We examined the relationships between the acoustic structure of fallow deer groans and male quality. We found that body size was negatively related to the minimum formant dispersion and not related to the fundamental frequency parameters of groans. We also found that minimum fundamental frequency and to a lesser extent, minimum formant dispersion, were related to dominance rank. Dominance was in turn strongly related to male mating success. The acoustic structure of sexually selected calls often contains information on different phenotypic traits of the caller that is potentially available to other individuals [4], [16], [24], [43]. Recent research has shown that both fundamental frequency-related and formant-related parameters are important in determining the individuality of fallow deer groans [41]. The results of the current study suggest that the same acoustic parameters also have the potential to reliably signal male fitness-related traits, and highlight a role for fundamental frequency (F0) in broadcasting information on social dominance previously only demonstrated in primates [4], [44].

Body size was not related to the fundamental frequency parameters of groans. This is similar to results for other mammals (rhesus macaques, *Macaca mulatta*, [18]; red deer, [16]; lions, *Panthera leo*, [45]; elephant seals, *Mirounga leonina*, [24]) and confirms that the growth of male larynx (and resulting fundamental frequency) is therefore at least partially dissociated from the growth of overall body size. In fallow deer, F0 is lower in males than in females [41], [46] and decreases as fawns mature [46]. Therefore, F0 variation still reflects sexual size dimorphism in the vocal apparatus and may be used to distinguish sex and possibly stage of development of the animal.

We found that body size was strongly negatively related to the minimum formant dispersion even though earlier research has shown that fallow bucks do not pull the larynx all the way down to its physiological limit during groaning (the sternum, [40]). Fallow deer males can groan more than 60 times per minute and call rate could be used as an assessment cue in competitive interactions [35], [47]. Thus, pulling down the larynx to an extent that is not maximal might be adaptive for fallow bucks if this allows senders to reach high calling rates when a correct indication of body size is still provided. Similarly to red deer, conveying information about body size through formant dispersion might play an important role in both agonistic interactions and mate attraction [38], [48], [49].

Body size was related to individual formants to a lesser extent than to the minimum formant dispersion (Dfmin). Body size was also more strongly related to higher formants (F5min and F6min) than to lower ones (F1min–F4min). These results clearly agree with those of other studies in which the relationships between individual body size and formants were mainly attributed to the higher formant frequencies and formant dispersion, because these reflect vocal tract length more precisely than lower ones [16], [18], [24], [50].

Higher ranking males produced groans with lower minimum fundamental frequency (F0min). The F0 of groans is relatively stable within individuals before the peak of the rut and accounts for a large proportion of vocal individuality [41]. Therefore, during the early phase of the rut, F0 is likely to represent a cue to the physical characteristics of the caller rather than to the motivational state or vocal effort of the animal. In humans (*Homo sapiens*), men with high androgen levels have voices with low F0 (pitch) and women preferred these males, especially close to ovulation [44], [51]. Sexual selection may also have favoured the evolution of vocal cues to the hormonal state (and therefore competitive ability) in fallow deer males, and led to the selection of lower pitch vocalisations indicating higher-quality individuals. Our results suggest that the acoustic parameters are indirectly related to mating success through social dominance. In humans, males with lower F0-voices have higher reproductive success and this is likely to be due to greater access to mates [52]. Therefore, in fallow deer, the negative relationships between the minimum fundamental frequency (F0min) and minimum formant dispersion (Dfmin) with mating success, are probably mediated by the dominance rank and body size of males, respectively [7]. These characteristics along with a large investment in vocal display are crucial in determining access to females and in turn mating success in fallow deer [7], [34]. We therefore suggest that females could use multiple cues from male vocal behaviour when trying to choose the best mate among those of similar quality.

The minimum formant dispersion (Dfmin) was only marginally related to dominance rank and body size was not related to rank. The sample size we used in our study was probably not sufficient to reveal an effect of body size on rank [7]. Assuming that a large body size is important for winning contests and therefore reaching high-ranking positions, males should use Dfmin as a cue to body size. This has been recently shown in red deer in which males perceive the differences in the Dfmin of roars produced by different competitors and use them to adjust their vocalisations and behaviour accordingly [38].

It is important to note that the majority of social dominance relationships between males were established through non-contact interactions before the rut and therefore before males became vocal [30]. During this time, males live in bachelor herds, and direct assessment of body size and body mass of the competitors is likely to play the major role in determining the outcome of the interactions and therefore the dominance ranks of males [7]. During the rut, the dominance relationships previously established are modified by fights, and males are expected to assess the status of their opponents [30]. Reliable assessment cues are those physically or physiologically linked to fighting ability and males may rely on several of these to assess each other during contests [5]. The use of acoustic cues reflecting the social dominance and therefore the overall resource holding potential of the individual, would then be crucial, especially when the opportunity for visual and olfactory assessment is poor such as at long distances and at night.

We found that dominance rank was the factor that was most strongly related to male mating success and body size appeared to play a secondary role. This result confirms that in fallow deer, as in several other ungulates, reaching a high ranking position is important for males to gain matings [53], [54], [55].

Minimum formant dispersion (Dfmin) and fundamental frequency (F0min) are among those variables that contain most of the information about individuality in fallow deer [41]. According to Dale et al. [56], traits signalling individuality should be characterised by different properties from those coding for male quality. However, some traits can have a role in both individual recognition and assessment of male competitive abilities [57]. Additional investigations together with playback experiments are needed to elucidate the independent role of the acoustic components of groans in conveying identity and quality assessment cues.

In conclusion, this study shows for the first time that the fundamental frequency (F0) of sexually-selected male vocalisations contains reliable information about social dominance in a non-primate species. Our study also confirms the role of formants in revealing male body size in mammals. F0 and formant frequencies may therefore represent acoustic cues to male quality that have primarily evolved in response to intrasexual selection. Other aspects of male vocal behaviour such as the long-term investment in vocal display [34], are instead likely to influence mate choice more directly in fallow deer.

## Materials and Methods

### Study Site and Population

The study was conducted on a herd of European fallow deer in Phoenix Park (53° 22′ N, 6° 21′ W), Dublin, Ireland. Since 1971 the majority of fawns were ear tagged each year in June, by the park authorities and others. All males used in this study were tagged, of known age and therefore individually recognisable.

### Morphological Measurements

We used body size measurements taken from 17 different males (five in 1996, one in 1997, one in 1999, three in 2001, six in 2002 and one in 2003). Males were caught immediately before (third week of September) or after the breeding season (third week of November). The males were sedated by a veterinary surgeon using a mixture of etorphine hydrochloride (18–20 µg/kg^−1^, C-Vet Veterinary Products) and xylazine (360–420 µg/kg^−1^, Rompun Dry Substance, Bayer), which was administered intramuscularly by gas-propelled darts. We measured a segment of one hind leg for each male using callipers and this was used as an indicator of skeletal size [7], [58]. Additional measurements (distance from the pre-orbital gland to the tip of the nose on both sides of the head) were also taken for a different study [40]. The handling time was generally less than 10 mins. Immobilisation was reversed by intravenously injection of an antidote containing a combination of antagonistic drugs, diprenorphine hydrochloride, (Revivon, 24–28 µg/kg^−1^, C-Vet Veterinary Products) and antipamozole hydrochloride (50 µg/kg, Antisedan, Pfizer) in a total volume of less than 2 ml. We then monitored the males until they were fully alert and they generally ran away from the area where the handling had been carried out. The males did not show any adverse effects as a result of the handling and many of them (n = 11) later gained matings during the rut. All procedures were approved by the University of Zurich and comply with the laws of Ireland.

### Observations

We conducted behavioural observations during the rut in 1997, 2000, 2002 and 2003. The rut refers to the period when matings occur. During this time, all-event recording of agonistic interactions and matings was carried out every day from dawn to dusk (circa 11 hours per day). There were 7–13 observers in the field at all times and the coverage of animals was maximised. The measure of male mating success was based on the number of observed copulations, and this provides a very good estimator of their reproductive success [59].

### Dominance Relationships

The outcomes of the agonistic interactions were used to calculate the dominance rank of each male by applying the David's score method [60]. This method is the most appropriate when interactions are recorded over a long period of time, because it takes into account repeated interactions between dyad members that may determine win/loss asymmetries [61]. Dominance ranks were calculated for males that interacted with at least 10% of other mature males.

### Recording and Selection of Groans

Recordings were made using a Sennheiser MKH 70 directional microphone connected to a Sony digital audio tape recorder DAT-TCD D100. Groans were recorded between dawn and sunset at a distance of 10 to 50 m from the vocalizing animal.

**Figure 3:**
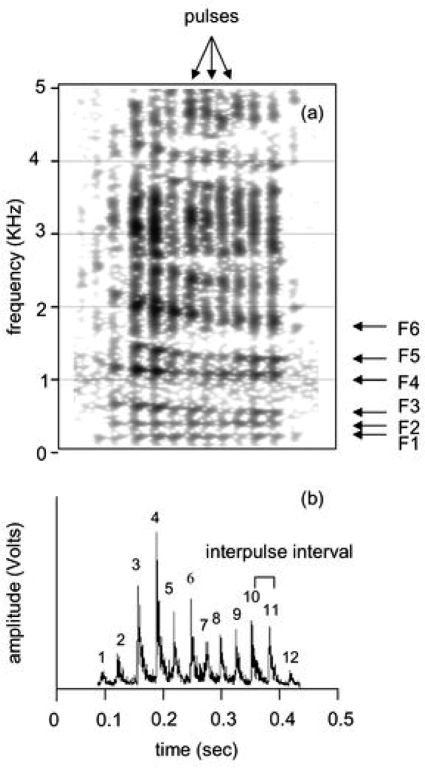
Narrow band spectrogram of a common groan and its relative envelope. On the spectrogram (a), the pulses and the first six formants are indicated. On the envelope of the signal (b), each peak of frequency is detected and indicated as “pulse”.

Vocalisations were imported into a computer using Avisoft-SASLab Pro 4.38 at a sampling rate of 22.05 KHz and saved in WAV format, and at 16-bit amplitude resolution [62]. The recordings that did not contain energy above 8 KHz were down-sampled to 16 KHz for a better frequency resolution. Narrow-band spectrograms of groans (Fig. 3a, FFT method, window length = 0.03 s, time step = 1000, frequency step = 250, frequency resolution = 20 Hz, Gaussian window shape, dynamic range = 35 dB) were edited using Praat 4.5.01 DSP package (P. Boersma and D. Weenink, University of Amsterdam, The Netherlands). Vocalisations with high levels of background noise were not considered for analysis.

During the breeding season, fallow deer males feed very little and lose approximately 26% of their body weight [63], and there is some evidence from primates and deer that the acoustic structure of vocalisations can be affected by exhaustion [4], [64]. We therefore analysed recordings taken between October 8 and October 20 when only a small proportion (15% or less) of the total number of matings had usually occurred [7], and the majority of agonistic interactions among males were non-contact displacements [30]. This minimised the possibility that variation in the phonic structure of groans could have occurred due to exhaustion of the animals.

For each male, we selected groans from different bouts that were recorded during one or more days. We included in our analysis males between five and eight years of age because they had reached their asymptotic size and were not undergoing changes associated with senescence. Moreover, this range of ages includes the males that account for the vast majority of matings [28].

### Acoustic Analysis

Groans are low-pitched vocalisations and therefore a pulse-train structure is generally visible in the spectrograms (Fig. 3a). The pulses represent the vibrations of the vocal folds and determine the fundamental frequency (F0) of the call. Fundamental frequency is equivalent to the inverse of interpulse interval and this can be measured as the distance between consecutive pulse onsets (Fig. 3b). Distances between pulses were measured automatically from the envelope (amplitude vs. time) of the signal by using Pulse Train Analysis (Fig. 3b; method = rectification+exponential decay; pulse detection = peak search with hysteresis; time constant = 1 ms; threshold = 0.1 V; hysteresis = 16–19 dB) in Avisoft-SASLab Pro 4.38. We calculated the values of the F0 along the groan and then averaged these values to obtain the mean F0 (F0mean). Because all groans showed at least a modest frequency inflection, the minimum and the maximum F0 (F0min and F0max) were also included in the analysis.

In the spectrogram of groans, six formants are evident as horizontal frequency bands (F1–F6 in Fig. 3a). The decrease of these formant frequencies along the groan reflects the elongation of the vocal tract occurring during vocalisation [40]. Formants were estimated using Linear Predictive Coding analysis (LPC), (Sound: To Formant (burg) command) in Praat 4.5.01 DSP package. By performing a single LPC analysis on each groan, higher formants (F4–F6) were better detected and therefore more accurately measured than lower formants (F1–F3). We therefore conducted a double or a triple LPC analysis on each groan in order to get the best estimations of all formants. We first carried out an LPC analysis (time step = 0.01–0.02 s, maximum number of formants = 3–4, maximum formant = 700–850 Hz, window length 0.07–0.26 s) to measure the frequencies of the first three formants (F1–F3). Then we performed a second LPC analysis (time step = 0.01–0.02 s, maximum number of formants = 6–7 maximum formant = 1800–2600 Hz, window length 0.07–0.26 s) to estimate the last three formant frequencies (F4–F6). When the sixth formant was not detected by the second LPC analysis, we conducted a third LPC analysis (time step = 0.01–0.02 s, maximum number of formants = 5–6 maximum formant = 1800–2600 Hz, window length 0.07–0.26 s). We calculated the minimum frequencies of the six formants (F1min–F6min) from each groan by averaging the values over the last part of the call when formants become flat. This is the time when the larynx is pulled down at the maximum extent. Finally, we also estimated the minimum spacing of the formants (known as minimum formant dispersion, (Dfmin), according to [16].

We analysed the vocalisations of 17 different males recorded during five breeding seasons between 1997 and 2004 (six in 1997, three in 2000, two in 2002, four in 2003, and two in 2004). Vocalisations analysed in relation to male dominance rank and mating success were carried out in the same year of the behavioural observations (1997, 2000, 2002, 2003). Recordings from 2004 were only analysed in relation to male body size because data on matings and agonistic interactions were not collected during that breeding season. For three males, vocalisations were recorded and body size measurements taken during the same breeding season. For most of the males (N = 14), recordings were performed one (N = 12) or two years (N = 2) before or after their body size measurements were taken. We assumed that the body size measurement that we used (hind leg length) did not change in fully grown mature males.

The low fundamental frequency that characterises fallow deer groans is still detectable and measurable on the spectrogram of groans recorded at more than 100 meters from the source (E. Vannoni, unpublished data). By contrast, formant frequencies are frequently lost or distorted in recordings taken in suboptimal conditions, such as when the microphone is far from the vocalizing animal or when it is not facing the microphone [62]. Therefore, because of the variety of the recording conditions, it was not always possible to measure the fundamental frequency and the formants on the same groans and for all males. As a result, sample sizes (number of groans and number of males) varied among analyses.

### Statistical Analysis

We used a general linear mixed effect model (GLMM) procedure fitted with residual maximum likelihood estimation (REML, lme function; [65] to investigate the effect of body size on the acoustic parameters of groans (F0-related parameters: 186 groans; 10.9±1.1 per individual; Formant frequencies and Dfmin: 144 groans; 8.5±0.8 per individual; N = 17). We conducted a univariate GLMM for all the acoustic parameters. Individual identity was fitted as random term so that we controlled for repeated measurements of the same individual. Body size was fitted as a fixed effect.

We used a model selection procedure [66] based on Akaike Information Criterion (AIC) to examine the relationships between the acoustic parameters and dominance rank and mating success of the males, while controlling for body size. Observational studies, such as ours, are better suited to the model selection than to null hypothesis testing [66]–[68]. All models were fitted with maximum likelihood implemented in the program R (ML, lm function; [65]. Pooling males across different years may potentially require standardization of male ranking positions and mating success. However, we did not standardize these variables for several reasons. First, we initially included “Year of recording” as a factor in all models but as it never had a significant effect, we did not considered it further in the analyses (E. Vannoni and A.G. McElligott, unpublished data). Secondly, standardized ranks have been used in a similar study and the results were not affected if relative ranks were replaced with absolute ranks [4]. Finally, as the ratio between the total number of matings and the number of mature males involved in the rut was relatively constant across different years (E. Vannoni and A.G. McElligott, unpublished data), it was not necessary to standardize the mating success.

The model selection technique identifies the model that best describes the structure in a data set among all a priori fitted models considered, controlling for the number of parameters (K), included in each model. Because each model is associated with a biological hypothesis, model selection identifies the hypothesis that is best supported by the data. We applied the AIC criteria adjusted for small sample size (AICc, [65]). This implies the selection of a few simple models that are most biologically meaningful [69]. Therefore, we limited our analyses to the factors that we considered most important on biological grounds. We included body size and dominance rank in the models because they are known to play crucial roles in male mating success in many mammals, including fallow deer [7], [70]. Among the measured acoustic parameters, we selected those that are most biologically meaningful [38], [41], [48]. The minimum fundamental frequency (F0min) has the highest degree of inter-individual variation, among the acoustic parameters used to describe the phonic structure of fallow deer groans [41]. It represents the lowest rate of vocal fold vibration and among the F0 parameters, is the only one to be physiologically constrained [17], [18]. The minimum formant dispersion (Dfmin) is constrained by the length of the vocal tract and is used by red deer males to assess competitors, and by females to choose their mates [18], [38], [48]. Moreover, both F0min and Dfmin are related to reproductive success in red deer [16].

We formulated two sets of candidate models. The fitted models for dominance rank (First set of models: model 1 to 5, Table 1) included the effects of body size, minimum fundamental frequency (F0min), and minimum formant dispersion (Dfmin). The fitted models for mating success (Second set of models: model 6 to 13, Table 1) included the effects of body size, dominance rank, F0min, and Dfmin. Then, we applied the model selection procedure based on AICc to each of the two sets. The value of AICc for a given model is a measure of the loss of information resulting from the use of the model to explain a particular pattern. Therefore, the model with the smallest AICc value is estimated to best fit the data set relative to other models considered [66]. When the difference between the AICc values of two models (ΔAICc) is less than 2 units, both models have support and can be considered competitive. Models with ΔAICc ranging from 3 to 7 have considerably less support by the data, whereas models with ΔAICc>10 are poorly supported and therefore very unlikely [66]. Akaike weights (*w_i_*) indicate the probability that a particular model is supported by the data among those included in the set of candidate models [66]. Akaike weights are normalized across the selected models to sum to one, and are interpreted as probabilities. For instance, an Akaike weight of 0.75 for a model, indicates that given the data, it has a 75% chance of being the best one in the set. For each model, we also calculated the evidence ratio, defined as the ratio between the Akaike weight of the best model and the Akaike weights of the competing model, to determine to what extent it is better than another. We used the likelihood-ratio tests (LRT) to compare nested models and to assess statistical significance of the factors. The LRT statistics follows a χ^2^-distribution with degrees of freedom equal to the difference in the number of parameters.

Data on dominance ranks and mating success were not available for three of the males for which we had body size measurements. Therefore, to investigate the relationships between acoustic parameters and dominance rank and mating success, we used data from 14 males (F0-related parameters: 156 groans, 11.1±1.3 per individual; Formant frequencies and Dfmin: 115 groans, 8.2±0.9 per individual). We log-transformed dominance rank and mating success to achieve normality. One unit was added to the mating success of all individuals before applying the log-transformation. In this way, we were able to transform the value of those individuals who did not get any matings (value = 0). All analyses were performed using R statistical software [71]. All tests were 2-tailed and factors were considered to have a statistically significant influence if *p*<0.05. All means are given with standard errors.
